# Case report: Thirty-year progression of an EMPF1 encephalopathy due to defective mitochondrial and peroxisomal fission caused by a novel *de novo* heterozygous *DNM1L* variant

**DOI:** 10.3389/fneur.2022.937885

**Published:** 2022-09-23

**Authors:** Charlène Lhuissier, Bart E. Wagner, Amy Vincent, Gaëtan Garraux, Olivier Hougrand, Rudy Van Coster, Valerie Benoit, Deniz Karadurmus, Guy Lenaers, Naïg Gueguen, Arnaud Chevrollier, Isabelle Maystadt

**Affiliations:** ^1^MitoLab Team, UMR CNRS 6015-INSERM U1083, Unité MitoVasc, SFR ICAT, Université d'Angers, Angers, France; ^2^Department of Histopathology, Royal Hallamshire Hospital, Sheffield, United Kingdom; ^3^Wellcome Centre for Mitochondrial Research, Faculty of Medical Sciences, Translational and Clinical Research Institute, Newcastle University, Newcastle upon Tyne, United Kingdom; ^4^GIGA-CRC in vivo Imaging, University of Liège, Liège, Belgium; ^5^Department of Neurology, CHU Liège, Liège, Belgium; ^6^Département d'Anatomie Pathologique, CHU Liège, Liège, Belgium; ^7^Division of Pediatric Neurology and Metabolism, Department of Pediatrics, Ghent University Hospital, Ghent, Belgium; ^8^Institut de Pathologie et de Génétique, Gosselies, Belgium; ^9^Service de Neurologie, Centre Hospitalier Universitaire d'Angers, Angers, France; ^10^Service de Biochimie et Biologie Moléculaire, CHU Angers, Angers, France; ^11^Faculté de Médecine, URPhyM, Université de Namur, Namur, Belgium

**Keywords:** mitochondrial fission, DRP1, *DNM1L*, encephalopathy, EMPF1

## Abstract

Mutations in *DNM1L* (*DRP1*), which encode a key player of mitochondrial and peroxisomal fission, have been reported in patients with the variable phenotypic spectrum, ranging from non-syndromic optic atrophy to lethal infantile encephalopathy. Here, we report a case of an adult female patient presenting with a complex neurological phenotype that associates axonal sensory neuropathy, spasticity, optic atrophy, dysarthria, dysphasia, dystonia, and ataxia, worsening with aging. Whole-exome sequencing revealed a heterozygous *de novo* variant in the GTPase domain of *DNM1L* [NM_001278464.1: c.176C>A p.(Thr59Asn)] making her the oldest patient suffering from encephalopathy due to defective mitochondrial and peroxisomal fission-1. *In silico* analysis suggested a protein destabilization effect of the variant Thr59Asn. Unexpectedly, Western blotting disclosed profound decrease of DNM1L expression, probably related to the degradation of DNM1L complexes. A detailed description of mitochondrial and peroxisomal anomalies in transmission electron and 3D fluorescence microscopy studies confirmed the exceptional phenotype of this patient.

## Introduction

*DNM1L* is located on chromosome 12p11.21 and encodes the 81.8 kDa human dynamin-1-like protein (DNM1L), formerly called dynamin-related protein 1 (DRP1). DNM1L protein belongs to the conserved family of dynamins and plays a key role in the regulation of mitochondrial and peroxisomal fission (1–5). As for other dynamins, DNM1L comprises a large GTPase domain, including the GTP-binding motifs that are needed for guanine nucleotide binding and hydrolysis, and two additional domains, the middle domain and the GTPase effector domain (GED), which are involved in oligomerization and regulation of the GTPase activity. DNM1L forms homodimers which are recruited to the outer membrane of the mitochondria and to the surface of peroxisomes, where they induce mitochondrial or peroxisomal fission, by wrapping around the scission site to constrict and sever the membrane through a GTP-hydrolysis-dependent mechanism (6). Excessive activation and upregulation of DNM1L has been found to be associated with aberrant mitochondrial fragmentation, causing neuronal damage in various neurodegenerative diseases, such as Alzheimer's disease, Parkinson's disease, Huntington's disease, and amyotrophic lateral sclerosis (7). Furthermore, a neural cell specific Drp1-/- mice model emphasized the role of Drp1 in embryonic forebrain development and synapse formation (8).

In humans, heterozygous *DNM1L* variants were reported in isolated autosomal dominant optic atrophy type 5 (OPA5, MIM#610708), and monoallelic or biallelic variants were associated with encephalopathy due to defective mitochondrial and peroxisomal fission (EMPF1, MIM#614388). Thirty-eight EMPF1 patients have been reported to date, showing a wide phenotypic spectrum and a variable degree of severity, ranging from early death to slowly progressive encephalopathy with hypotonia and developmental delay (9–11), while seizures were inconstantly reported. Dystonia, dysarthria, spasticity, microcephaly, insensitivity to pain, peripheral neuropathy, ataxia, optic atrophy, and brain anomalies (dysmyelination, corpus callosum anomalies, and T2 hyperintensities mainly in the cortex and the basal ganglia) are common clinical features associated with *DNM1L* variants.

Here, we report the oldest EMPF1 patient, whose clinical presentation recapitulates the entire phenotypic spectrum of *DNM1L*-related encephalopathy, except for the microcephaly and brain MRI anomalies, emphasizing the pejorative evolution of the clinical presentation. Heterozygous mutations in the GTPase domain usually affect *DNM1L* enzymatic activity without affecting its abundance, but here the p.(Thr59Asn) variant induced the full loss of *DNM1L* normal forms probably following the degradation of the DNM1L complexes. We confirmed, with a detailed description of mitochondrial and peroxisomal anomalies in transmission electron and 3D fluorescence microscopy studies, the abnormal cellular features of this patient.

## Case report

The 32-year-old female patient is the only child of healthy non-consanguineous Caucasian parents (Supplementary Figure 1). Pregnancy was uncomplicated. She was born at 38-week gestation by cesarean section due to narrow maternal pelvis, with a weight of 2,950 g (P45), height of 47.5 cm (P35), and normal head circumference (precise measure not known). Neonatal adaptation was correct (APGAR score 9 at 1 min). During the first month, sucking difficulties and poor weight gain were noticed. The patient showed hypotonia and global developmental delay. She walked autonomously at 24 months, with a tendency to tiptoe and with ataxic gait, despite intensive physiotherapy. She progressively developed a tetrapyramidal syndrome. A diagnostic hypothesis of cerebral palsy was suggested, but CT scan revealed no brain anomalies. She spoke the first intelligible words at 3.5 years and built sentences at 4 years. During childhood, she had behavioral problems, such as tantrums, which decreased with aging. She had high pain tolerance. She had learning difficulties and required a school for special educational needs. At the age of 7, global IQ was estimated around 66 at the Wechsler scale. She can read and write, but mathematics remains a challenge. Since age 10, her neurological status deteriorated with increasing gait disturbances, dysarthria, and dysphagia. At the age of 12 years, clinical examination revealed a scoliosis, which was treated with a brace. She also had an ophthalmological follow-up for nystagmus and bilateral optic atrophy (Table 1). Her puberty was delayed, with the first menstrual cycle beginning at 16 years. At the age of 18 years, she developed tonic–clonic epileptic generalized seizures. Epilepsy was well controlled with lamotrigine treatment. At age 27, sleep study revealed severe dyssomnia, with fragmented sleep and a periodic limb movement disorder, treated with clonazepam.

**Table 1:** Summary of the clinical and genetic features of the patient in this study compared with EMPF1 patients described with a *de novo* heterozygous mutation in the GTPase domain of DNM1L.

	**Trujillano et al. (12)**	**Whitley et al. (13)**	**Verrigni et al. (14)**	**Longo et al. (15)**	**Keller et al. (9)**	**Liu et al. (10)**	**Wei et al. (16)**	**This study**
Mutation	c.607G>A p.(Val203Ile)	c.95 G>C p.(Gly32Ala)	c.668G>T p.(Gly223Val)	c.436G>A p.(Asp146Asn)	c.115A>G p.(Ser39Gly)	c.116G>A p.(Ser39Asn)	c.445G>A p.(Gly149Ar)	c.176C>A p.(Thr59Asn)
Inheritance	*de novo*	*de novo*	*de novo*	*de novo*	*de novo*	*de novo*	*de novo*	*de novo*
Gender	female	female	female	female	male	male	male	female
Age	8 months	7 years	6 years	5 years	10 years	3 years	4.5 years	32 years
Early developmental delay	+	+	-	+	+	+	+	+
Hypotonia	NA	+	-	++	+	+	+	+
Spasticity	+	+	NA	+	-	+	-	++
Dystonia	+	NA	NA	NA	+	NA	NA	+
Seizures	-	-	++ (>2.5 y)	-	+ (>8 y)	-	-	**+** **(>18 y)**
Peripheral neuropathy	+ (unspecified)	+ (sensory)	NA	++ (axonal, sensory)	+ (axonal, sensory)	NA	+ (axonal, sensory)	++ (axonal, sensory)
Feeding difficulties/failure to thrive	+	+	NA	+	++	NA	NA	+
Ataxia	NA	+	+	+	NA	NA	+	+
Nystagmus	+	+	NA	+	-	NA	-	+
Optic nerve atrophy	-	+	NA	-	-	NA	NA	+
Facial dysmorphism	NA	+	NA	NA	+	NA	-	+
Microcephaly	NA	+	NA	-	+	+	NA	**-**
Brain MRI anomalies	Abnormal myelination, cerebral white matter atrophy	-	T2 hyperintensities in the posterior white matter regions and in the cortical areas. Global cerebral and cerebellar atrophy	-	Left cerebellar venous angioma (non specific)	T2 hyperintensity of basal ganglia, ventriculomegaly	Delayed myelination, thin corpus callosum	**-**
Scoliosis	NA	+	NA	-			NA	**+**
Other	Areflexia, Tongue fasciculations, Craniosynostosis, Chorea, Recurrent respiratory infections, Bilateral vocal cord paralysis	aphthous ulcers			Mitral valva insufficiency		Strabismus, mild cognitive impairment, dysarthria, equinous foot deformity	Insensitivity to pain, dysarthria, delayed menarche

At clinical examination at 29 years, the patient's height was 155 cm (P3) and weight was 42 kg (−3SD). Her head circumference was 54.5 cm (P55). She had a long and asymmetric face with deep-set eyes, divergent strabismus, high nasal bridge, and high arched palate (Supplementary Figure 1). Standard X-ray showed scoliosis that was 35 degrees with a Risser index of 4. Neurological examination revealed spastic quadriparesis with brisk tendon reflexes, a pseudobulbar syndrome with dysarthria and dysphagia, dystonia, ataxia, and lower limbs proprioception deficit.

Currently, at 32 years, the patient daily presents with drop attack of the lower limbs, without loss of consciousness. Spasticity, contractures, ataxia, peripheral neuropathy, dysarthria, and dysphagia are worsening with aging. She uses a wheelchair and requires assistance with activities of daily life, but can still feed herself. Her treatment consists in lamotrigine, coenzyme Q10, clonazepam, sotalol, alprazolam, and levetiracetam. She is receiving speech therapy, physiotherapy, occupational therapy, and psychological support.

Brain MRI, performed in childhood, was controlled at adulthood and remained unremarkable. A fluorodeoxyglucose (FDG)-positron emission tomography (PET) revealed basal ganglia hypermetabolism compared to cerebral cortex. At adult age, lumbar puncture showed normal intracranial pressure and normal glucose and cells in cerebrospinal fluid. Metabolic screening (plasma and urinary amino acids, very-long-chain fatty acid) was unremarkable, except for increased lactate:pyruvate and 3-hydroxybutyrate:acetoacetate ratios, which were more pronounced post-prandially (lactate 2.41 mmol/L, nl 0.4–1.8; L:P 18.5, nl <15; 3OHB:AA 3.5, nl <1). At 23 years, a severe axonal sensory neuropathy was detected in the four limbs by nerve conduction studies (NCS).

## Genetic investigations

The patient underwent serial genetic investigations (molecular karyotyping, *POLG* sequencing, spino-cerebellar-ataxia/Friedreich/dentatorubral-pallidoluysian triplet expansions analyses) that were unremarkable. Clinical exome sequencing was performed on DNA extracted from the whole blood of the patient and her parents, with Illumina NextSeq technology, according to the manufacturer's protocol (Supplementary material and method). In the proband, a heterozygous *DNM1L* c.176C>A p.(Thr59Asn) (Chr12(GRCh38): g.32701488 C>A) variant was detected (NM_001278464.1). The ClinVar accession number is SCV001780462. The variant was confirmed by PCR and direct Sanger sequencing. The primer sequences and reaction conditions are available upon request. This variant is not listed in genomic variant database (GnomAD). Paternity and maternity were molecularly confirmed, and the variant was not found in the parents suggesting that it arose *de novo* (paternal coverage 146X; maternal coverage 99X) (Supplementary Figure 2). The pathogenicity of the variant was assessed *in silico* using multiple prediction software, with results strongly supporting his deleterious and damaging effect, classified as class 5 variant by the American College of Medical Genetics ACMG prediction program (PS2, PS3, PP2, PP3, PP5). DNA next-generation sequencing did not detect any additional variant nor CNV on the second *DNM1L* allele (coverage 100% >20X). Moreover, no additional candidate causal variant was identified by clinical exome (in particular, no mutation was detected in the *OPA1* gene with a coverage of 99.95% >20X). In the proband, cDNA short- and long-range amplification and sequencing revealed that the mutant and the wide-type *DNML1* alleles are both expressed, in similar proportion (50%-50%), in blood extracted lymphocytes, in lymphoblastoid cells, and in fibroblasts (Supplementary Figure 2 and Supplementary material and method).

In addition, mitochondrial genome sequencing performed with Illumina MiSeq technology showed the m.10254G>A p.(Asp66Asn) variant in *MT-ND3* (NC_012920) at 1.5% heteroplasmy in blood, at 9.9% heteroplasmy in urine, and at 1.3% heteroplasmy in skeletal muscle. This variant has no frequency in MitoMap and is classified as pathogenic in ClinVar (accession number VCV000155887). In the mother, this mitochondrial variant was found at 1.3% heteroplasmy in urine but was not significantly detected in blood (Supplementary Figure 2).

## Biochemical and histological characterizations

**Figure 1:**
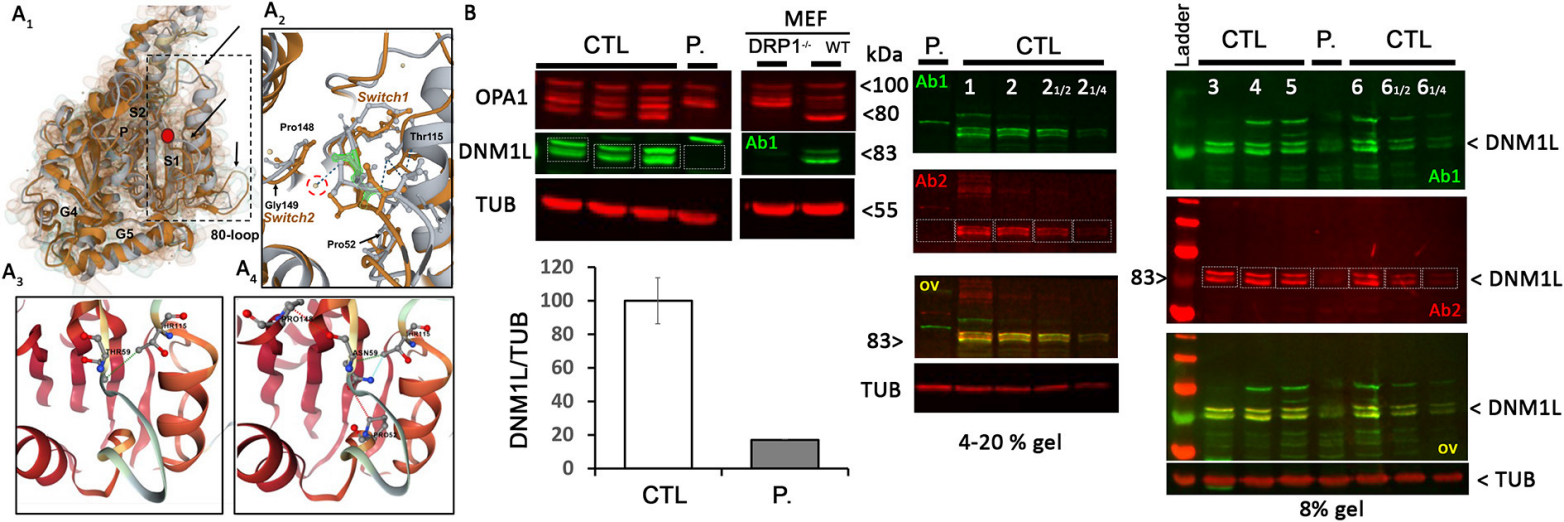
DNM1L/DRP1 immunostaining shows the loss of *DNM1L* normal forms in patient fibroblasts. **(A**_**1**_**)** Comparison of the GTPase domain structure of nucleotide-free, wild-type (orange, pdb code 4H1U) and mutated THR59ASN DNM1L (gray) in ribbon and Gaussian volume representation, using RCSB pairwise structure alignment tool. The mutation site is indicated by a red circle. Modeling indicates a disorganization of the switch 1 in which the mutation is located, but also in the unique 80-loop, αE1^G^ helix and the loop connecting αE1^G^ and the β2A sheet (indicated by black arrow). **(A**_**2**_**)** Close-up view of the switch1 in the nucleotide-free structure of the WT (orange) and mutated (gray) DNM1L. ASN59 is underlined in green. Water molecules are represented in yellow. The catalytic water molecule connecting switch I (THR59) with switch II (GLY149) by hydrogen bonds is circled in red. This interaction is lost with the amino acid switch THR59ASN. Amino acids interacting with THR59 or ASN59 are indicated. Detailed view of amino acid interactions in nucleotide-free wild-type **(A**_**3**_**)** and mutated **(A**_**4**_**)** protein structures. Hydrophobic interaction is in green, hydrogen bonds in red, and VDW in blue. Analysis was performed using Dynamut2. **(B)** OPA1 and DNM1L/DRP1 protein quantification and α-tubulin (TUB) as loading control in control (CTL) and patient (P.) fibroblasts. Control and DRP1 knock out MEFs: Mouse embryonic Fibroblasts models. On the right, overlay yellow staining of 2 DNM1L/DRP1 antibodies Ab1 and 2 indicates specific staining. ≪ $\frac{1}{2}$ ≫ and ≪ $\frac{1}{4}$ ≫: controls were charged at 1/2 and $\frac{1}{4}$ to control the linearity range of DNM1L signal detection.

The Thr59Asn substitution is located in the GTPase domain, within a stretch of highly conserved residues of the switch I essential for the ligand binding, the catalytic activity of the enzyme, and the interaction with other domains, such as the switch 2 (5). *In silico* structural modeling of DNM1L Thr59Asn and comparison with the wild-type, nucleotide-free structure (PDB:4H1U) indicated conformational changes in the switch s1, αE1^G^ helix, and two disorders loop, the 80-loop and the loop connecting αE1^G^ and the β2A sheet (Figure 1A). In addition, bioinformatics tests predicted that the Thr59Asn variant affects the stability of DNM1L (Supplementary Figure 3). While contradictory results were obtained according to the predictive tools used for the nucleotide-free structure, the Thr59Asn substitution is predicted to destabilize the protein in its nucleotide-bound state, particularly in the GDP-bound one (Supplementary Figure 3B). We characterized the skin fibroblasts from the patient carrying the DNM1L p.Thr59Asn mutation to explore its pathogenic nature. Unexpectedly, by Western blot, we showed a strong decrease of DNM1L protein expression (Figure 1B), with the very faint of signal corresponding to the two 81-83Kda bands generally revealed by DNM1L immunostaining. In a process that remains to be understood, the mutated DNML1 form would complex with the wide-type DNML1 form and be degraded. Interestingly, in the patient fibroblasts, the profile of OPA1 isoforms is also different, although no mutation or splicing alteration was detected by NGS and long-range *OPA1* RNA studies (data not shown).

**Figure 2:**
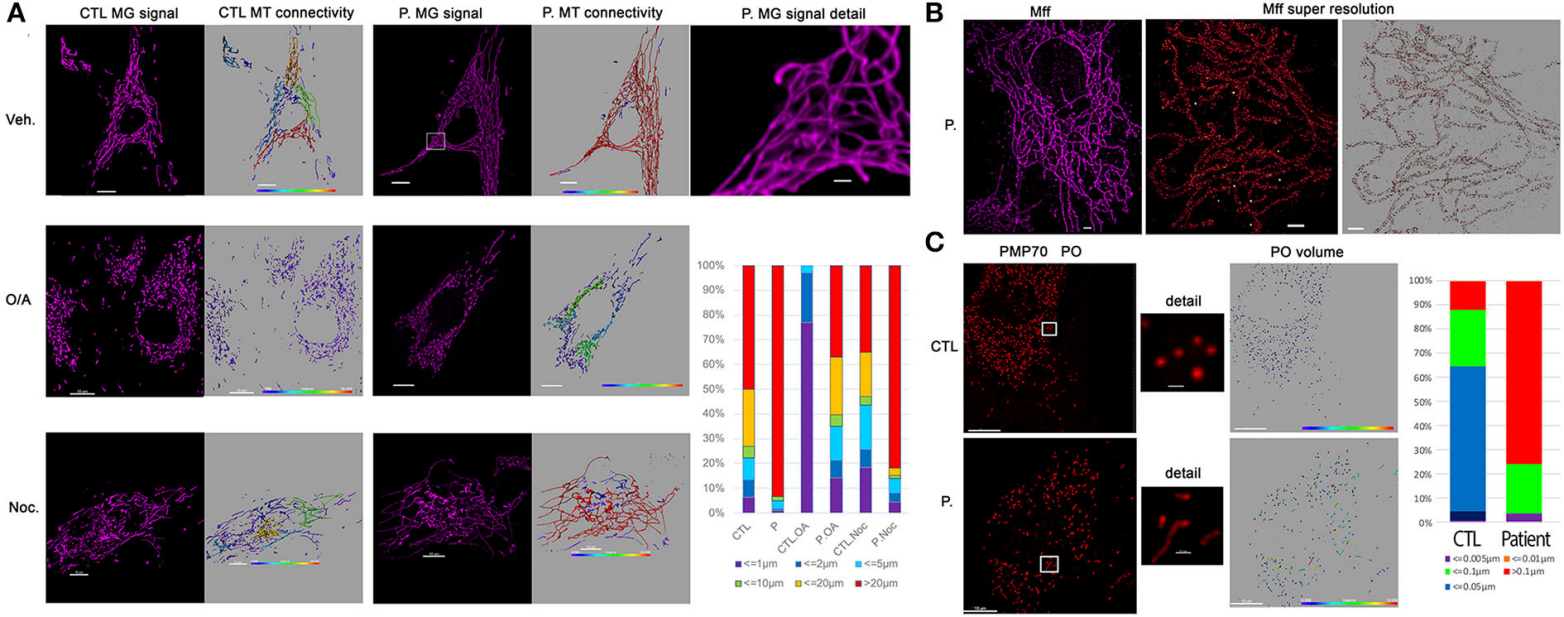
Patient fibroblasts show highly connected mitochondrial network and elongation of peroxisome compare to control. **(A)** Representative fluorescent images of mitochondrial network structure (in purple) in control (CTL) and patient (P.) fibroblasts. Mitochondrial volume was assessed using the MitoTracker Green fluorescent signal which was analyzed using Imaris software. The color code highlights the different lengths of the mitochondrial tubules with short isolated mitochondria in violet and connected network in red. To present the changes in mitochondrial morphology in patient's cells, types of mitochondria were classified into six groups according to mitochondrial volume, for example, red color represents mitochondria > 20 μm^3^. Oligomycin 4 μg/ml coupled to antimycin 2 μg/ml (O/A) treatment leads to mitochondrial fission within 4 h in CTL cells. 6 h nocodazole (Noc), 10 μM disrupts microtubules. **(B)** Immunostaining against mitochondrial Mff DRP1 receptor. Representative TIRF image of mitochondria (left, in purple) shows the hyper-connectivity of the network. Single-molecule localization microscopy dSTORM was used to analyze mitochondrial MFF distribution. The white dots are the fiducial signals used to control drift during acquisition. Scale bar: 2 μm. **(C)** Immunostaining against peroxisomal PMP70 protein. Peroxisome (PO) volume was assessed using fluorescent signal by Imaris software and color-coded. To present the changes in peroxisome morphology in patient's cells, types of Peroxisomes were classified into five groups according to peroxisome volume, for example, red color represents peroxisomes > 20 μm^3^. Scale bar = 10 μm; Scale bar in details 1 μm.

As loss of DNM1L function leads to a block in mitochondrial and peroxisomal fission, we conducted microscopic organelles' imaging by fluorescent MitoTracker Green probe and immunostaining against the peroxisomal membrane protein 70, respectively. Normal skin fibroblasts show a set of dynamic tubular networks with some isolated mitochondria that we could fragment by bioenergetic stress (Figure 2A). 3D analysis and morphometric measurements revealed a hyper-connected mitochondrial network (Figure 2A) without any isolated mitochondria. After antimycin/oligomycin treatment, which induces a mitochondrial stress by the inhibition of complexes III and V, the mitochondrial network remained tubular with some swollen elements. As hyper-fusion requires mitochondria to be brought together, the microtubule network was chemically disrupted using nocodazole to prevent mitochondrial movements. Following cytoskeletal depolymerization, the mitochondrial network remained abnormally connected in the patient's fibroblasts suggesting an absence of fission. We performed immunostaining of Mff, the mitochondrial outer membrane DRP1 receptor, by super-resolution microscopy and confirmed normal Mff distribution on mitochondrial hyper-connected network of the mutated fibroblasts (Figure 2B). Compared to control fibroblasts, disclosing small and abundant peroxisomes, mutated fibroblasts showed a decreased number of peroxisomes, which appeared significantly elongated (Figure 2C). These results strongly suggest a defect in DRP1 function, with downstream consequences on mitochondrial fission and peroxisomal shape in the DNM1L p.Thr59Asn fibroblasts.

**Figure 3:**
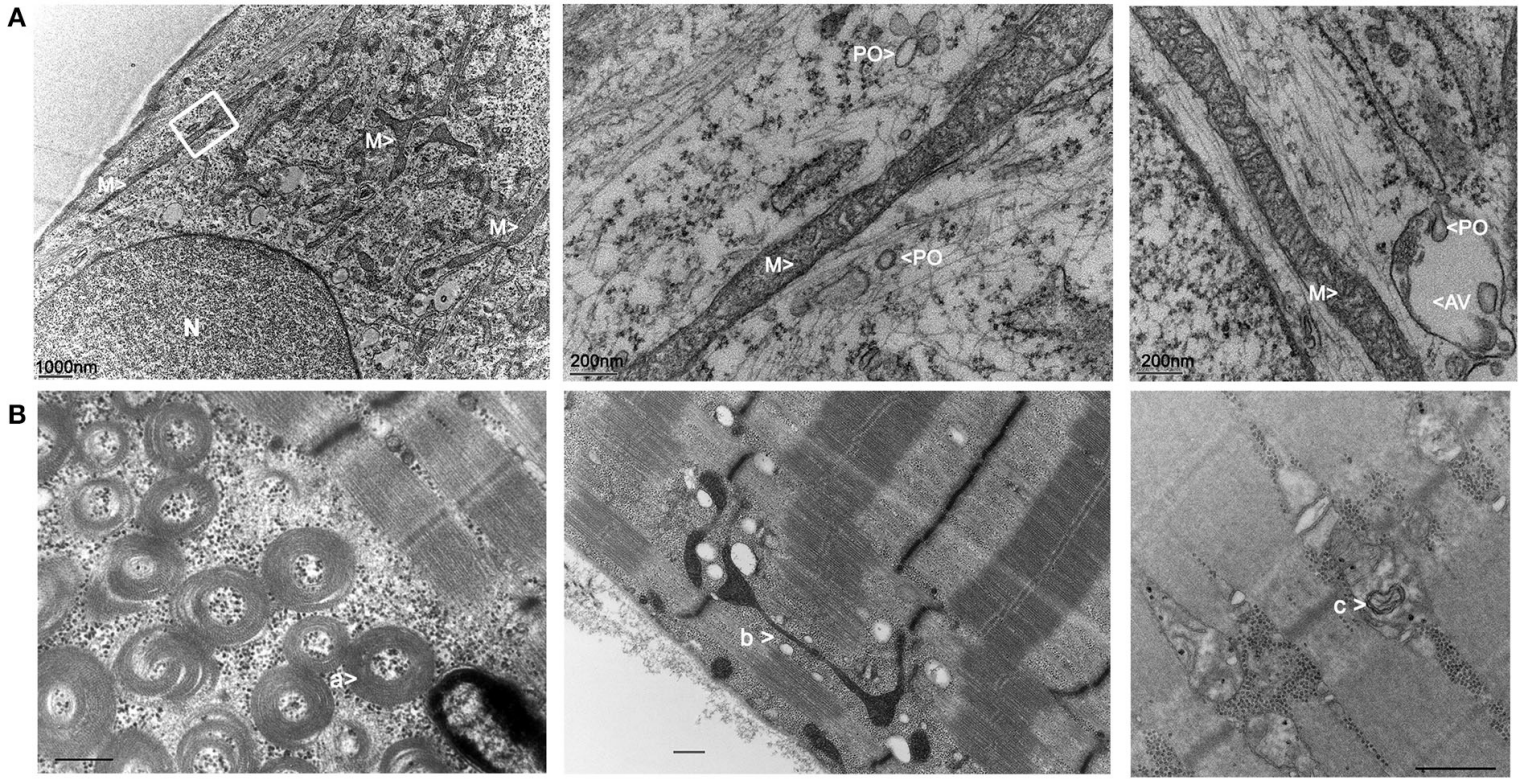
Electron microscopy imaging of the patient fibroblasts and muscle. **(A)** Skin fibroblasts: M, mitochondria; PO, peroxisomes; N, nucleus; AV, autophagic vacuole. **(B)** Muscle biopsy: a: concentric laminated bodies; b: mitochondrial nano-tunnel; c multiple degenerative myelinoid intramitochondrial foci.

Transmission electron microscopy (TEM) pictures of patient fibroblasts confirmed the presence of enlarged peroxisomes (17) with many peroxisomes in autophagic vacuoles and longer mitochondria than in control cells (Figure 3A). Immunohistological studies of a muscle biopsy revealed no specific anomaly, and only one ragged red fiber was detected. However, muscle TEM disclosed elongated mitochondria, with long slender protrusions from some mitochondria, likely corresponding to mitochondrial nano-tunnels. No ultrastructure defect was evidenced, with normal cristae distribution, apart from a couple of mitochondrial profiles with some degenerated cristae. Subsarcolemmal concentric laminated bodies and several multiple degenerative myelinoid intramitochondrial foci were identified (Figure 3B). No respiratory chain complex enzymatic deficiency was shown in muscle and skin biopsy.

## Discussion

Here, we report the oldest EMPF1 patient, whose clinical presentation recapitulates the entire phenotypic spectrum of *DNM1L*-related encephalopathy, except for the microcephaly and brain MRI anomalies (Table 1). This patient shows a complex neurological phenotype associating developmental delay, progressive spastic quadriplegia, dystonia, dysarthria, dysphagia, axonal neuropathy, ataxia, nystagmus, optic atrophy, epilepsy, low sensitivity to pain, scoliosis, delayed menarche, and learning difficulties. Peripheral neuropathy, usually described as axonal with predominant sensory loss, is constantly reported in EMPF1 patients harboring a heterozygous DNM1L variant in the GTPase domain. This peripheral neuropathy is progressive, as confirmed by electroneuromyography examinations of the patient, which showed a drastic decrease of the sensory nerve action potentials and worsening of muscle denervation signs between ages 7 and 23. Interestingly, delayed menarche described in our patient may also be part of the *DNM1L*-related phenotypic spectrum as it was already reported in another 27-year-old female harboring a *de novo* heterozygous *DNM1L* variant in the GED domain (18).

The first case of lethal EMPF1 encephalopathy due to defective mitochondrial and peroxisomal fission was described in 2007, in a new-born girl harboring a *de novo* heterozygous mutation in DRP1 middle domain, and presenting with microcephaly, abnormal brain development, optic atrophy, persistent lactic acidemia, and mildly elevated plasma concentration of very-long-chain fatty acids (19). Additional descriptions of severe lethal forms of EMPF1 associated with *de novo* heterozygous variants located in DRP1 middle domain were reported (9, 11, 20–22). Outside the DRP1 middle domain, *DNM1L* variants were also reported in the GTPase domain, initially at a heterozygous state associated with isolated autosomal dominant optic atrophy and at compound heterozygous state associated with autosomal recessive EMPF1 (23). More recently, several patients suffering from encephalopathy due to defective mitochondrial and peroxisomal fission-1 were described with *de novo* heterozygous variants located in the GTPase domain, as observed in our patient (9, 10, 12–16) (Table 1). Interestingly, most of the mutated amino acids in this latter cohort (Ser39, Asp146, Gly149) were reported as the most relevant residues of the five GTP-binding stretches, as the Thr59 residue mutated in our patient (5).

A dominant-negative mechanism is classically hypothesized for heterozygous mutations located in the GTPase domain, as they affect the enzymatic domain of the protein without altering the capacity of the protein to homopolymerize, leading to a loss of function of the wild-type protein within the homopolymers (24). This dominant-negative effect was also suggested by the functional experiments on DNM1L/DRP1 (Thr59Asn) mutant cells and model organism studies (2, 5, 25, 26). However, the present study revealed an almost complete loss of DNM1L protein in the mutated fibroblasts, which suggests the degradation of the mutated and non-mutated DNM1L proteins. As the complete loss of DNM1L/DRP1 in mice is embryonic lethal, our results raise questions concerning DNM1L/DRP1 complex instability within different tissues and its downstream consequences on this patient's clinical presentation.

In addition, the phenotype of our proband might be influenced by the heteroplasmic presence of the m.10254G>A p.(Asp66Asn) variant in *MT-ND3*. This variant was reported at 90% heteroplasmy in the muscle sample of a patient presenting with a severe Leigh syndrome associated with early developmental regression and ophthalmoplegia (27). However, the 1.3% heteroplasmy level measured in muscle sample of our patient is very low, and no necrotic lesion in the brainstem, basal ganglia, or thalamus, and characteristic of the Leigh syndrome was observed by brain MRI. Consequently, the contribution of this mitochondrial variant to the DNM1L patient clinical presentation is most likely not preponderant.

EMPF1 phenotype overlaps with the clinical spectrum of other diseases linked to proteins involved in mitochondrial dynamics fusion (MFN2, OPA1) and fission (MFF, MID49/MIEF2, DNM2). EMPF1 is clinically very similar to Behr's syndrome (OMIM #210000) due to biallelic *OPA1* mutations (28) and to EMPF2 (OMIM #614785) due to biallelic *MFF* mutations (29). Mitochondrial morphology is maintained through the opposing forces of fission and fusion, and this balance is crucial in muscles and neurons. Interestingly, functional studies have already demonstrated the essential role of threonine-59 in Drp1. In *C. elegans DNM1L/DRP1* ortholog gene, the analogous Thr61Asn mutation has a strong dominant-negative effect on mitochondrial morphology, by inhibiting the mitochondrial outer membrane fission (25). Moreover, in COS-7 cells transfected with the DNM1L/DRP1 (Thr59Asn) allele, the mitochondrial network invariably collapsed into large perinuclear aggregates, composed of clustered mitochondrial tubules, reflecting a defective distribution of the fused mitochondrial network throughout the cell (26).

In this respect, reconstruction of 3D fluorescence microscopy images showed a hyper-connected mitochondrial network in the patient fibroblasts, compared to control, and a defect of fragmentation in mitochondrial stress context. This was well described in *DNM1L*-related optic atrophy or encephalopathy, contrasting with hyper-fragmentation observed in *OPA1*-related optic atrophy (14, 15, 19, 30). In muscle, the long slender double-membrane protrusions from mitochondria likely correspond to nano-tunnels that connect the matrices of non-adjacent mitochondria, which might emphasize immobilized mitochondria “in distress” (31). These protrusions are observed in normal tissue but are more abundant in presence of mtDNA mutations (32). Similarly, TEM analyses of the patient muscle revealed the presence of concentric laminated bodies that are common to other mitochondrial myopathies (33). Altogether, these peculiar observations reflect mitochondrial stress in our patient and should be confirmed in future by additional muscle sample analyses of EMPF1 patients.

DNM1L protein also mediates peroxisomal fission. Peroxisomes are dynamic and multifunctional organelles involved in the beta-oxidation of the very-long-chain fatty acids and in the formation of plasmalogen, which is an important component of the myelin. Very-long-chain fatty acid dosages performed in our DNM1L/DRP1 (Thr59Asn) patient showed normal results (data not shown), while our data revealed a decreased number of significantly elongated peroxisomes, as already reported for other DNM1L patients (15). Additional studies demonstrated that DNM1L/DRP1 is recruited by PEX11 to the peroxisome membranes to induce their division and that the dominant-negative DNM1L/DRP1 (Thr59Asn) mutant reduces peroxisome abundance (2). More recently, X-ray structure analyses of DNM1L confirmed that threonine-59 is located in the switch I motif and is essential for the GTPase reaction involved in mitochondrial and peroxisome segregations (5). The great impact of the p.(Thr59Asn) mutation on peroxisomal network and shape is thus a central contribution to the severity of the clinical phenotype reported here (15, 19, 24).

This work evidences a novel *de novo* heterozygous *DNM1L* mutation causing an original adult presentation of encephalopathy due to defective mitochondrial and peroxisomal fission-1 EMPF1. Structural anomalies of mitochondria and peroxisomes observed in the primary cell culture from skin biopsy attest the main impact of *DNM1L*/DRP1 (Thr59Asn) mutation on mitochondrial and peroxisomal dynamics, which has to be correlated with the original post-translational modifications of DNM1L and OPA1, emphasizing the complexity of DRP1 regulation and its role in causing EMPF1 pathogenicity.

## Data availability statement

The datasets presented in this study can be found in online repositories. The name of the repository and accession number can be found below: National Center for Biotechnology Information (NCBI) CinVar, https://www.ncbi.nlm.nih.gov/clinvar/, VCV001195874.1, (accessed May 6, 2022).

## Ethics statement

The studies involving human participants were reviewed and approved by Institutional Review Board Committee of the University Hospital of Angers Authorization number: AC-2012-1507. The patients/participants provided their written informed consent to participate in this study. Written informed consent was obtained from the individual(s) for the publication of any potentially identifiable images or data included in this article.

## Author contributions

IM, CL, and AC: drafting of the manuscript. IM, CL, AC, NG, and GL: revision of the manuscript. IM and AC: study concept or design. GG, AC, VB, DK, RV, OH, CL, and NG: acquisition of data. AC, CL, VB, BW, AV, and CL: interpretation of data. All authors contributed to the article and approved the submitted version.

## Funding

This work was supported by “Université d'Angers.” AV is supported by a Henry Wellcome Fellowship (215888/Z/19/Z).

## Conflict of interest

The authors declare that the research was conducted in the absence of any commercial or financial relationships that could be construed as a potential conflict of interest.

## Publisher's note

All claims expressed in this article are solely those of the authors and do not necessarily represent those of their affiliated organizations, or those of the publisher, the editors and the reviewers. Any product that may be evaluated in this article, or claim that may be made by its manufacturer, is not guaranteed or endorsed by the publisher.
