# Supplementary material for: Case report: Thirty-year progression of an EMPF1 encephalopathy due to defective mitochondrial and peroxisomal fission caused by a novel de novo heterozygous DNM1L variant
Source: Front Neurol. 2022 Sep 23;13:937885. doi: 10.3389/fneur.2022.937885 (PMC9538651; doi:10.3389/fneur.2022.937885)
Supplement: Supplementary file 1 [file Data_Sheet_1.PDF]

Supplementary Materials and Methods

DNA Next-Generation Sequencing

Library preparation was performed using the SeqCap EZ library prep kit from Roche.

Briefly, after concentration determination, DNA was mechanically sheared (COVARIS), 3'extremities were repaired and adenylated and finally, indexing adaptors were ligated.

Amplification and purification steps allowed to get the pre-capture libraries.

For enrichment, samples have been pooled (8 per capture) and target sequences have been isolated via a custom probes kit from Roche aimed to specific capture of more than 4500 disease-associated genes or a commercial kit targeting mitochondrial genes.

Targeted regions were simultaneously sequenced on a Nextseq or a MiSeq sequencing machine (Illumina, San Diego, CA) for clinical exome or mitochondrial genes analysis respectively. Reads alignment, variants calling and sorting have been performed through our in-house bioinformatics pipeline.

Regarding the clinical exome, 20X coverage has been reached for >98.2% of the targeted regions and mean coverage was 288X. Trio analysis was performed and HPO criteria's were used in order to select genes involved in those symptoms (HP:0000639 Nystagmus; HP:0001138 Optic neuropathy; HP:0001250 Seizures; HP:0001251 Ataxia; HP:0001257 Spasticity; HP:0001260 Dysarthria; HP:0001263 Global developmental delay; HP:0001328 Specific learning disability; HP:0001332 Dystonia; HP:0001347 Hyperreflexia; HP:0002360 Sleep disturbance; HP:0002650 Scoliosis; HP:0007024 Pseudobulbar paralysis; HP:0010831 Impaired proprioception; HP:0011283 Abnormality of the metencephalon; HP:0011442 Abnormality of central motor function; HP:0030880 Raynaud phenomenon). Using this phenotypic filter and a 2% maximal allelic frequency in ExAC database, 207 variants have been sorted out (in coding regions and proximal intronic regions, from -14 to +6). Sanger sequencing using a standard protocol was performed for validation.

Regarding mitochondrial genes analysis, the same bioinformatics pipeline has been applied. A mean coverage of 1948X with >99% of targeted genes covered at 1000X has been reached on DNA from urine sample. In muscular DNA, the mean coverage was 1000x higher and reached 19129X, all the genes being fully covered at 1000X.

mRNA/cDNA studies

Cells extracted from blood sample, lymphoblastoid cell line, lymphoblastoid cell line treated with puromucin (6h) and fibroblasts culture, were conserved in Tripure solution at -80°C. RNA was extracted using Maxwell RSC miRNA Plasma and Serum Kit (Promega), with a Maxwell RSC48 instrument (Promega), following the manufacturer's recommendations. RNA was prepared for cDNA libraries using a M-MLV Reverse transcriptase kit (Life Technologie, Invitrogen), following the manufacturer's recommendations. Long-range amplifications (exons 1-9 and exons 1-20) were performed using standard protocols (available on request).

Cell Culture

To obtain lymphoblastoid cell line, mononuclear cells extracted from patient's peripheral total blood were infected in vitro with Epstein Barr Virus (EBV). Patient and control primary fibroblasts were derived from skin biopsies after obtaining written consent. Amplifications were performed from passage 1 to 5, allowing cell cryoconservation of 8 samples per cell line. Fibroblasts were cultured in Dulbecco's minimum essential medium in the presence of 10% foetal calf serum (FCS), 1% uridine and 1% pyruvate, in a humidified atmosphere (95% air, 5% CO2) at 37◦C. Monolayers of primary fibroblast were routinely maintained in T75 flasks. All experiments were conducted on cells with similar passage numbers, ranging from 6 to 20, in order to avoid artefacts due to senescence. Cells were treated with dimethylsulfoxide (Sigma) vehicles, Oligomycine 4µg/ml (PubChem SID 24898006), Antimycine 2µg/ml (PubChem SID 24891355), 4 hours or 10 µM Nocodazol (PubChem CID4122),6 hours.

Electron microscopy

The samples used for transmission electron microscopy were processed using standard protocols (Cassereau *et al,* 2008). Human skin Fibroblasts were fixed in 2.5% electron microscopy-grade glutaraldehyde (LFG Distribution, Sainte Consorce, France) in 0.1 M phosphate buffer pH 7.4 for 16 h at 4 °C. Samples were rinsed with 0.1 M phosphate buffer and post-fixed by incubation with 1% osmium tetroxide and 1% potassium ferricyanide in H_2O for 60 min at room temperature. Samples were then rinsed with distilled water, dehydrated in graded series of ethanol solutions and finally embedded in Epon at 60 °C for 48 h. Embedded samples were cut into 60 nm-thick sections, which were contrast-stained with 3% uranyl acetate in ethanol 50° for 15 min and then observed under a Jeol JEM 1400 transmission electron microscope operating at 120 keV and equipped with a Gatan Orius digital camera. Analysis and measurements were performed on 10 cells. Peroxisomes were

identified by the presence of a single membrane, a homogenous fine-granular matrix, and profile size. Mitochondria were identified by the presence of a double membrane with the inner membrane shaping folding in the matrix space. Autophagic vacuoles were identified by the presence of a single membrane containing partially digested organelles and cytoplasmic materials.

Western Blotting

Cellular proteins were solubilized in a Laemmli buffer, and 40 µg was resolved by SDS-PAGE in a 8% acrylamide gel. Proteins were then transferred to a nitrocellulose membrane in a semi-dry transfer apparatus (iBlot 2 Dry blotting system, ThermoFisher, France). Mouse anti-OPA1 (BD Biosciences, 612607), mouse anti-DRP1 ([3B5] ab56788, Abcam), Rabbit anti-DRP1 ([EPR19274] ab184247, Abcam), and Mouse Anti-alpha Tubulin ([DM1A] ab7291, Abcam) primary antibodies were used (1:1000 dilution). Membranes were incubated for two hours in the dark, with anti-mouse and anti-rabbit coupled, respectively, with Alexa Fluor 680 and Alexa Fluor 790 dyes (ab186694 and ab186697 Abcam, 1:10,000 dilution). Membranes were washed twice with TBS 1X–Tween 0.1% and once with TBS 1X. Fluorescence was detected at 700 and 800 nm with an Odyssey XF imaging system (LI-COR Biosciences, Bad Homburg, Germany). Band intensities were quantified with Image Studio software (LI-COR Biosciences, Bad Homburg, Germany).

3D Fluorescence microscopy

Cells were incubated for 15 minutes with 100 nM Mitotracker® green (Molecular Probes) to stain mitochondrial network. For fluorescence imaging, coverslips were mounted in housing and placed on the stage of an inverted wide-field microscope ECLIPSE Ti-E (Nikon) equipped with a 100x oil immersion objective (Nikon Plan Apo100x, N.A. 1.45) and an Andor NEO sCOMS camera controlled by Metamorph® 7.7 software (Molecular Devices, Sunnyvale, CA, USA). A precision, piezoelectric driver mounted underneath the objective lens allowed faster Z-step movements, keeping the sample immobile while shifting the objective lens. 31 image planes were acquired along the Z-axis at 0.1 µm increments. For mitochondrial network characterization, acquired images were iteratively deconvolved using Huygens Essential® software (Scientific Volume Imaging, Hilversum, The Netherlands), with a maximum iteration scored 50 and a quality threshold at 0.01. Imaris 8.0® software (Bitplane, Zurich, Switzerland)

was used for 3D processing and morphometric analysis. The mitochondrial networks were modelled in 3D, and thresholds were defined in order to classify mitochondria depending on their volume (Imaris isosurface tools).

Immunofluorescence

Human skin fibroblasts were seeded at a density of ~90,000 cells per well in a six-well plate containing 20-mm coverslips and incubated overnight. Cells were fixed with 4% paraformaldehyde (PFA) in PBS for 15 min. After fixation, cells were quickly washed 3 times in PBS and then cells were incubated in the blocking buffer (BF; PBS with 5% BSA) for 15 min. Coverslips were then washed in PBS three times and were incubated overnight with primary antibody diluted in the BF at 4°C on a rocking platform providing a gentle “wave” effect. Coverslips were then washed in BF three times for 5 min and subsequently incubated for 90 min at room temperature with goat anti-mouse Alexa 647 IgG (H+L) secondary antibody diluted in BF in dark chamber. Finally, coverslips were washed in PBS two times for 5 min, and keep in PBS at 4 degrees up to the assembly for the STORM acquisition. Immunodetection of mitochondria was achieved on fixed fibroblasts using antibodies against MFF Rabbit PolyAb – proteintech – 17090.

STORM Acquisition

For super resolution imaging, the cavity of a clean single depression slide (Paul Marienfeld, Lauda-Königshofen, Germany) was filled with 50 µL of switching buffer (Abbelight, Paris, France), and covered by the coverslip, the sample side facing downward. The device was placed on the stage of an inverted motorized microscope NIKON ECLIPSE Ti-E (Nikon Instruments Europe, Amsterdam, The Netherlands) equipped with a CFI SR APO TIRF 100X ON1.49 objective, a Perfect Focus System, and a total internal reflection fluorescence (TIRF) ILas2 module (Roper Scientific, Martinsried, Germany).

Acquisition of images were proceeded using Metamorph 7.7 software (Molecular Devices, CA, USA). Image sequences were acquired with a single-photon sensitive camera Evolve 128TM EMCCD 512 x 512 imaging array, 16 x 16 µm pixels (Photometrics, Tucson, AZ, USA). Acquisitions were performed at fixed temperature 25°C in a dark heating chamber (Okolab NA, Pozzuoli, Italy). Phase contrast was first used for orientation and focus adjustment. Prior to STORM imaging, a multichannel TIRF fluorescence microscopy image was acquired for

subsequent comparison with STORM image. Images were acquired with an integration time of 60 ms per frame. The total acquisition time points for each sequence were adapted to the observed structure and to the labelling density (5,000 to 20,000 frames). Images were analyzed and reconstructed using the WaveTracer module integrated into Metamorph software (Kechkar 2013).

Structural modelling of the Wt and mutated DNM1L.

The monomer structure of Wild-type (Wt) and of Thr59Asp DNM1L variants were generated using both Phyre2 software (Protein Homology-fold Recognition server; www.sbg.bio.ic.ac.uk/phyre2/) and SWISS-MODEL server (http://www.expasy.org/spdbv; Swiss Institute of Bioinformatics, Geneva, Switzerland; GMQE : 0.71 ; QMEANDisCo Global: 0.69 ± 0.05). Obtained PDB files for Wt and mutated DNM1L 3D structures were loaded and compared on RCSB PDB Pairwise Alignment server (https://www.rcsb.org/alignment). Mutated DNM1L 3D structure was further superposed and compared to existing structural data (free- nucleotide DNM1L, PDB 4BEJ and 4HIU) using both SWISS-MODEL and RCSB PDB Pairwise Alignment servers. The 4BEJ crystal structure lack amino acids surrounding the mutation site, i.e. within the Switch 1 (Chain cut between residues Gly54 and Val58), the Switch 2 (Chain cut between residues Ile118 and Val 125 and between residues Lys152 and Asp161) and the 80-loop (Chain cut between residues Ser71 and VAL85), therefore the 4H1U structure (Wenger et al., 2013) was privileged for the analyses.

The ligand present in crystal structures 4H1V (GMP-PNP, non-hydrolyzable GTP analog; Wenger et al., 2013) and 3W6P (GDP-AIF4; https://www.rcsb.org/structure/3W6N) cannot be transferred by homology in the model due to the change in amino acid in the conserved domain of interaction with these ligands. Therefore, predictions of change in protein stability were favored for nucleotide-bound DNM1L analyses.

Homodimers were generated using the GalaxyWeb tool GalaxyHomomer (http://galaxy.seoklab.org/cgibin/submit.cgi?type=HOMOMER) based on the pdb files of the monomeric structures obtained from Phyre2 and the sequence of the mutated protein.

Calculating changes in protein stability

Four different protein stability programs were used to predict the effects of missense mutations on the stability of *DNM1L* protein: DUET (Pires et al., 2014).

http://structure.bioc.cam.ac.uk/duet), mCSM (Pires et al., 2014b). http://structure.bioc.cam.ac.uk/mcsm), PopMuSIC (Dehouck et al., 2011). https://soft.dezyme.com), and Dynamut2 (Rodrigues et al., 2021). http://biosig.unimelb.edu.au/dynamut2). Features and performances of DUET, PopMuSIC and Dynamut are described in (Marabotti et al., 2021). mCSM relies on graph-based signature concept and predicts not only the effect of single-point mutations on protein stability, but also protein–protein and protein–nucleic acid binding (Capriotti et al., 2006).

Statistical analysis :

At least three biological replicates of each fibroblast cell line were analyzed. The values of mutant cell lines compared to control cells that were beyond #2 SD (standard deviation) to the mean were considered to be significantly different.

References :

Capriotti E, Calabrese R, Casadio R. Predicting the insurgence of human genetic diseases associated to single point protein mutations with support vector machines and evolutionary information. Bioinformatics. 2006 Nov 15;22(22):2729-34. doi: 10.1093/bioinformatics/btl423. Epub 2006 Aug 7. PMID: 16895930.

Cassereau J, Chevrollier A, Gueguen N, Malinge MC, Letournel F, Nicolas G, Richard L, Ferre M, Verny C, Dubas F, Procaccio V, Amati-Bonneau P, Bonneau D, Reynier P. Mitochondrial complex I deficiency in GDAP1-related autosomal dominant Charcot-Marie-Tooth disease (CMT2K). Neurogenetics. 2009 Apr;10(2):145-50. doi: 10.1007/s10048-008-0166-9. Epub 2008 Dec 17. PMID: 19089472.

Dehouck Y, Kwasigroch JM, Gilis D, Rooman M. PoPMuSiC 2.1: a web server for the estimation of protein stability changes upon mutation and sequence optimality. BMC Bioinformatics. 2011 May 13;12:151. doi: 10.1186/1471-2105-12-151. PMID: 21569468; PMCID: PMC3113940.

Kechkar A, Nair D, Heilemann M, Choquet D, Sibarita JB. Real-time analysis and visualization for single-molecule based super-resolution microscopy. PLoS One. 2013 Apr 30;8(4):e62918. doi: 10.1371/journal.pone.0062918. PMID: 23646160; PMCID: PMC3639901.

Marabotti A, Del Prete E, Scafuri B, Facchiano A. Performance of Web tools for predicting changes in protein stability caused by mutations. BMC Bioinformatics. 2021 Jul 5;22(Suppl 7):345. doi: 10.1186/s12859-021-04238-w. PMID: 34225665; PMCID: PMC8256537.

Pires DE, Ascher DB, Blundell TL. DUET: a server for predicting effects of mutations on protein stability using an integrated computational approach. Nucleic Acids Res. 2014 Jul;42(Web Server issue):W314-9. doi: 10.1093/nar/gku411. Epub 2014 May 14. PMID: 24829462; PMCID: PMC4086143.

Pires DE, Ascher DB, Blundell TL. mCSM: predicting the effects of mutations in proteins using graph-based signatures. Bioinformatics. 2014 Feb 1;30(3):335-42. doi: 10.1093/bioinformatics/btt691. Epub 2013 Nov 26. PMID: 24281696; PMCID: PMC3904523.

Rodrigues CHM, Pires DEV, Ascher DB. DynaMut2: Assessing changes in stability and flexibility upon single and multiple point missense mutations. Protein Sci. 2021 Jan;30(1):60-69. doi: 10.1002/pro.3942. Epub 2020 Sep 11. PMID: 32881105; PMCID: PMC7737773.

Wenger J, Klinglmayr E, Fröhlich C, Eibl C, Gimeno A, Hessenberger M, Puehringer S, Daumke O, Goettig P. Functional mapping of human dynamin-1-like GTPase domain based on x-ray structure analyses. PLoS One. 2013 Aug 19;8(8):e71835. doi: 10.1371/journal.pone.0071835. PMID: 23977156; PMCID: PMC3747075.
