# Supplementary material for: Case report: Thirty-year progression of an EMPF1 encephalopathy due to defective mitochondrial and peroxisomal fission caused by a novel de novo heterozygous DNM1L variant
Source: Front Neurol. 2022 Sep 23;13:937885. doi: 10.3389/fneur.2022.937885 (PMC9538651; doi:10.3389/fneur.2022.937885)
Supplement: Supplementary file 2 [file Data_Sheet_2.PDF]

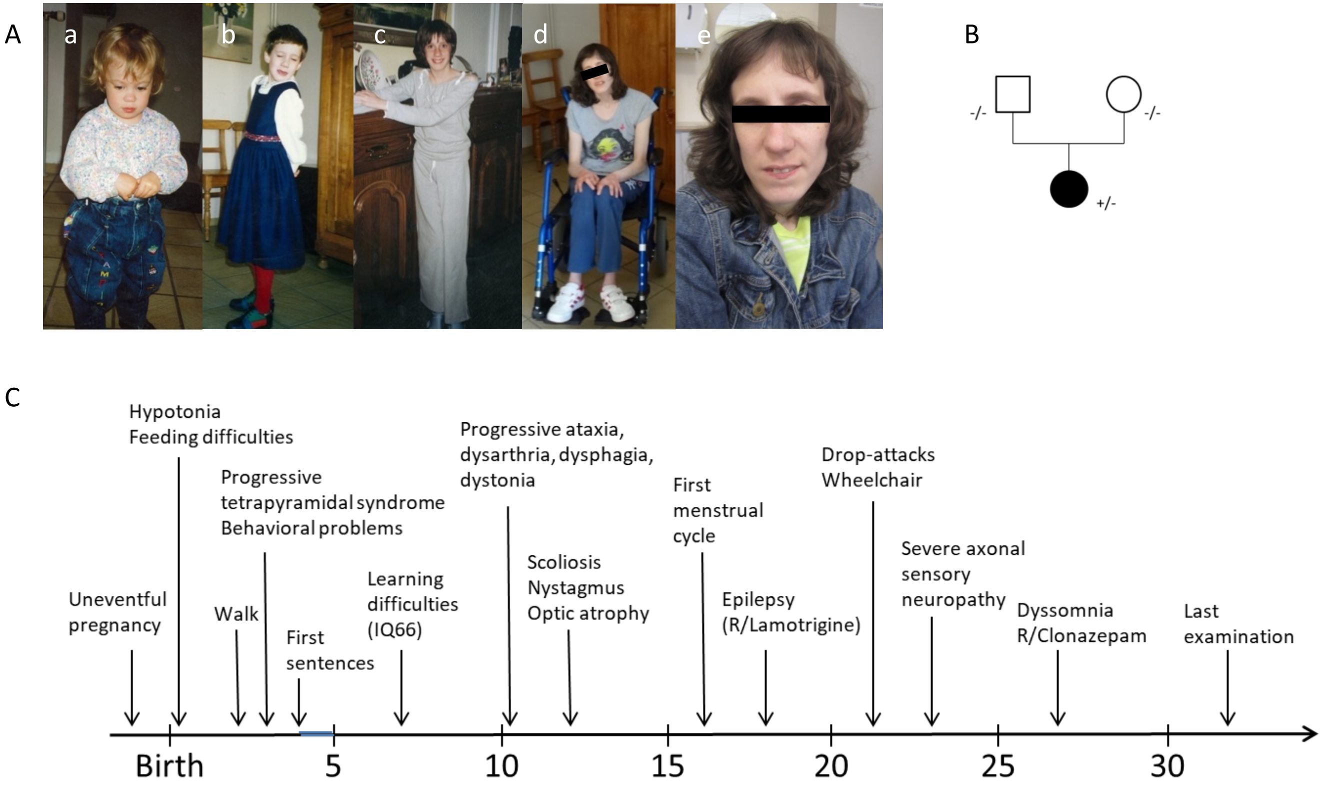


Supplementary Figure 1 :

(A) **Patient at the age of 2 years (a), 9 years (b), 15 years (c) and 32 years (d-e).** We note the progressive spastic quadriparesis. At adult age, the face is long and asymmetric, with deep-set eyes, divergent strabismus, high nasal bridge and high arched palate.

(B) **Genealogy** showing that the proband is the only child of nonconsanguineous parents.

(C) **Timeline** summarizing the clinical evolution of the patient.

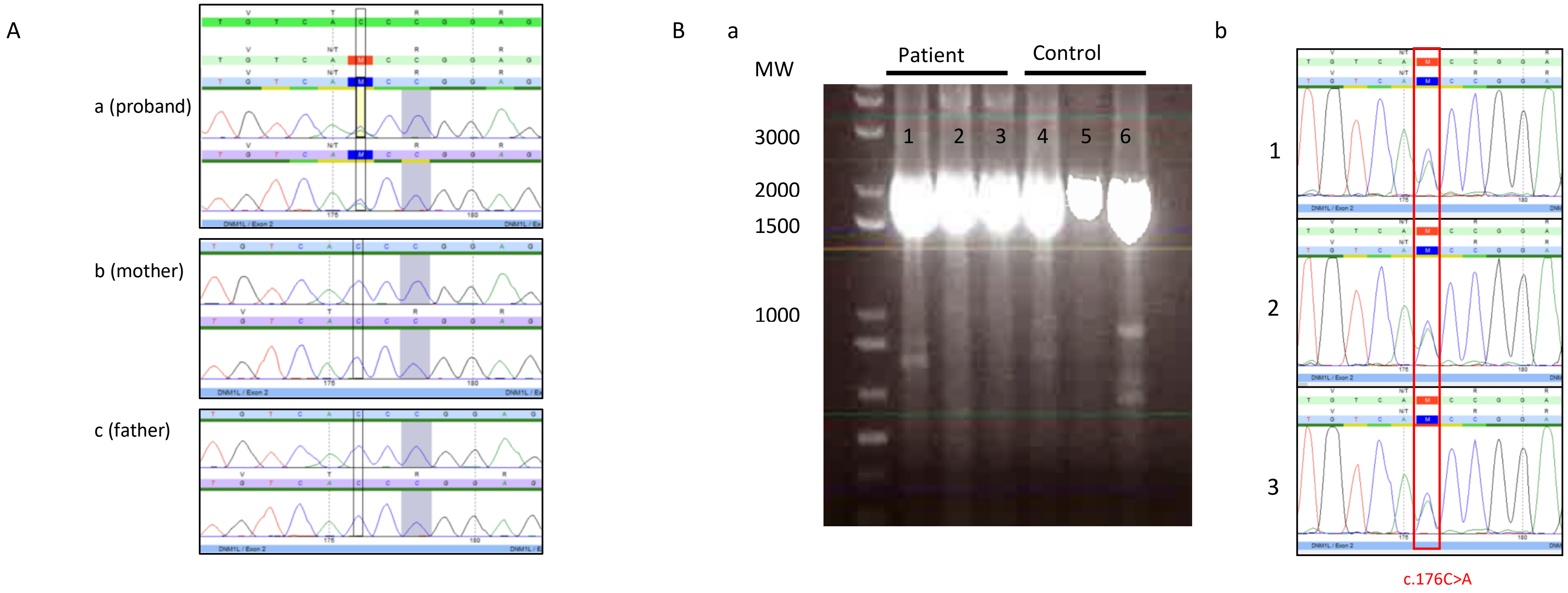


C

| | Samples | Mutant reads | Total reads | % heteroplasmy |
|---|---|---|---|---|
| Proband | Blood | 72 | 4871 | 1.5% |
| | Urine | 192 | 1947 | 9.9% |
| | Muscle | 244 | 18582 | 1.3% |
| Mother | Blood | 5 | 3066 | 0.16% |
| | Urine | 244 | 18582 | 1.3% |

Supplementary Figure 2 :
(A) **Sanger chromatograms** confirming the heterozygous status of our patient for the *DNM1L* c.176C>A p.(Thr59Asn) mutation (a) and the absence of the mutation in her parents (b-c). NGS analysis did not reveal parental mosaicism (maternal coverage 99X, paternal coverage 146x) (data not shown).
(B) ***DNM1L* cDNA amplification and sequencing.** (a) PCR long-range amplification (exons 1-20, 2225bp) showed no abnormal transcript in the patient (1-3) compared to control (4-6), in blood extracted lymphocytes (1, 4), in lymphoblastoid cells (3,6), and in lymphoblastoid cells treated with puromycin during 6h (2,5). (b) In the proband, sequencing analysis showed a similar expression of mutant and wide-type alleles in blood extracted lymphocytes (1), in

lymphoblastoid cells (3), and in lymphoblastoid cells treated with puromycin during 6h (2). Similar results were obtained in fibroblasts treated or not with puromycin (data not showed).

(C) **Mitochondrial DNA sequencing by NGS** in the proband and the mother**,** showing various heteroplasmy levels for the m.10254G>A p.(Asp66Asn) variant in *MT-ND3* in blood, urine and muscle samples.

Supplementary Figure 3

A

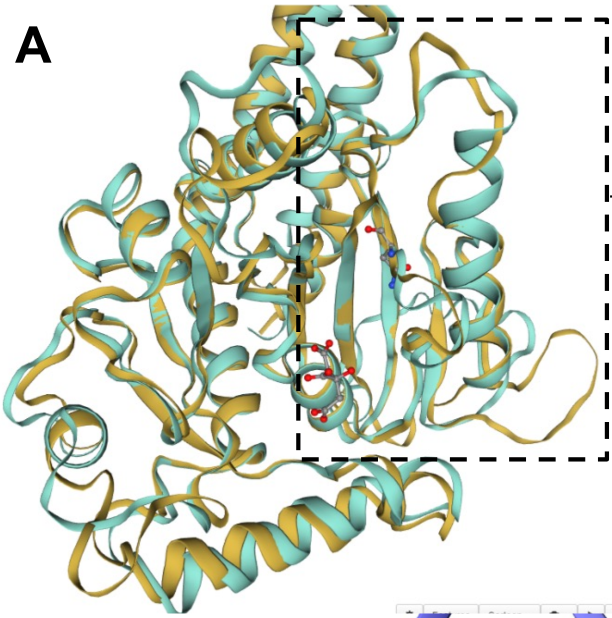

C

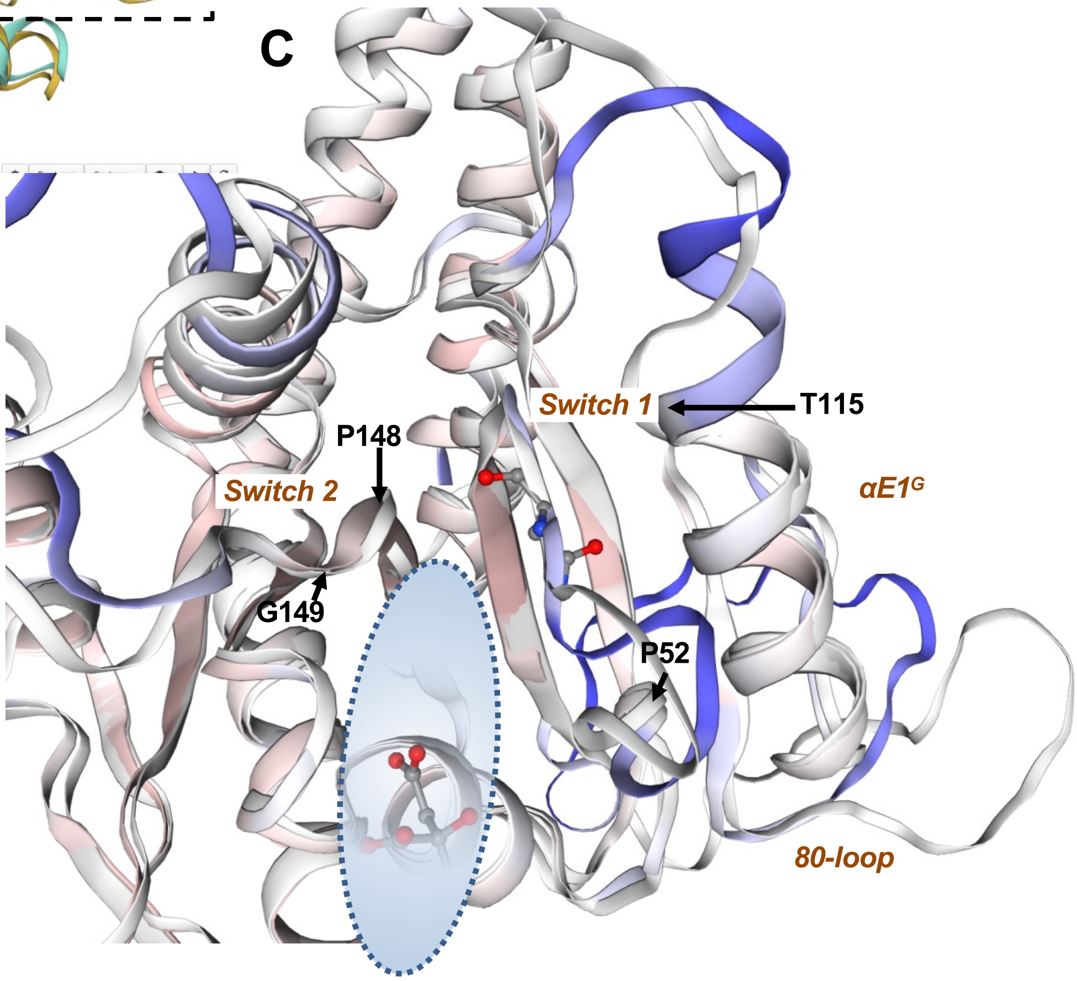


**GTPase domain**

B

| | | 4BEJ nucleotide-free | 4H1U nucleotide-free | 4H1V GMP-Pnp | 3W6P GDP |
|---|---|---|---|---|---|
| DUET | *ΔΔG* | N/A | -0.310 | -0.225 | **-0.787** |
| PopMusic | *ΔΔG* | **0.860** | **0.820** | **-0.600** | -0.500 |
| Dynamut2 | *ΔΔG* | -0.250 | **-0.610** | **-0.590** | **-0.720** |
| mCSM | *ΔΔG* | -0.222 | **-0.559** | **-0.569** | **-1.014** |
| mCSM-lig log(affinity fold chage) | | | | **-2.133** | **-1.712** |

A. **Stability of nucleotide free structure**. Upper panel, superposition of the nucleotide-free wild-type (orange, pdb code 4H1U) and mutated THR59ASN (light blue) structure in ribbon representation, using SWISS-MODEL comparison tool. Lower panel, close-up view of the GTP binding domain. Switch1, switch 2 and 80-loop are indicated in orange. Amino acids interacting with T59 or N59 (see supplemental Figure 1) are also indicated. Structure is represented according to change in B-factor range, as an indicator of gain in rigidity (blue) / flexibility (red). Comparison highlighted an increased rigidity (blue) in the switch 1 loop and αE1G helix and slight increased flexibility in core β sheets. Nucleotide binding site is indicated by the light blue oval.
B. **Evaluation of stability changes** due to Thr59Asn variant of the nucleotide-free (PBD:4BEJ and 4HIU) and nucleotide-bound DNM1L (PBD:4H1V, GMP.Pnp, a non-hydrizable GTP analog, bound and PDB:3W6P, GDP.AIF4 bound) using three different protein stability prediction programs. Decrease in ΔΔG indicated a destabilization effect. Variation of ΔΔG >0,5 was considered significant (in red).

Note that the 4BEJ crystal structure lacks amino acids surrounding the mutation site, i.e. within the Switch 1 (Chain cut between residues Gly54 and Val58), the Switch 2 (Chain cut between residues Ile118 Val 125 and between residues Lys152 and Asp161) and the 80-loop (Chain cut between residues Ser71 and VAL85).
